# Sujiaonori-Derived Algal Biomaterials Inhibit Allergic Reaction in Allergen-Sensitized RBL-2H3 Cell Line and Improve Skin Health in Humans

**DOI:** 10.3390/jfb8030037

**Published:** 2017-08-29

**Authors:** Nlandu Roger Ngatu, Mamoru Tanaka, Mitsunori Ikeda, Masataka Inoue, Sakiko Kanbara, Sayumi Nojima

**Affiliations:** 1 Graduate School of Health Sciences and Nursing, University of Kochi, Kochi 781-8515, Japan; mikeda@cc.u-kochi.ac.jp (M.I.); inoue@cc.u-kochi.ac.jp (M.I.); kanbara@cc.u-kochi.ac.jp (S.K.); nojimas@cc.u-kochi.ac.jp (S.N.); 2 Department of Nutrition, University of Kochi, Kochi 781-8515, Japan; m-tanaka@cc.u-kochi.ac.jp

**Keywords:** allergy, β-hexosaminidase, Sujiaonori, transepidermal water loss (TEWL), ulvan

## Abstract

Sujiaonori, a river alga growing in the Kochi prefecture, Japan, contains several bioactive compounds such as sulfated polysaccharides (ulvans), ω-3 fatty acids, and vitamins. Dietary intake of this alga-based supplement has been reported to increase circulatory adiponectin, a salutary hormone that is reported to be associated with healthy longevity and prevents a number of cardiovascular and metabolic disorders. This report highlights the anti-allergic and skin health enhancing effects of Sujiaonori-derived ulvan (Tosalvan) and supplement, respectively. RBL-2H3 cell line was used to investigate the anti-allergic effect of algal SP through the evaluation of β-hexosaminidase activity. Algal sulfated polysaccharides or SP (Tosalvan, Yoshino SP) were extracted from powders of dried alga samples provided by local food manufacturers. Report on the effect of daily dietary intake of Sujiaonori-based supplement on skin health is part of a four-week clinical investigation that, in comparison with a supplement made of 70% corn starch powder and 30% spinach powder mixture (twice 3 g daily), explore the beneficial effects of Sujiaonori algal biomaterial (SBM; 3 g taken twice daily) on cardiovascular, gastrointestinal and skin health in a sample of Japanese women. Transepidermal water loss (TEWL) was the skin health marker used in this study and was measured with the use of a corneometer. Significant reduction of β-hexosaminidase activity was observed in Tosalvan and Yoshino SP-treated cells (vs. control; *p* < 0.05), whereas dietary intake of SBM markedly reduced TEWL level after four weeks of supplementation, as compared to baseline TEWL (*p* < 0.001). Additionally, SBM improved TEWL better than the control product (*p* < 0.001). Findings contained in this report suggest that Sujiaonori-derived Tosalvan and Yoshino SP have anti-allergic potential and that the dietary intake of SBM has a beneficial effect on skin health.

## 1. Introduction

Marine algae have recently been used as source of innovative biomaterials that serve in the development of cosmetic products [1]. They are well-known as source of health-promoting bioactive materials such as ω-3 fatty acids, essential amino acids, sulfated polysaccharides, and vitamins A, B, C, and E that are used in cosmetic products development [2,3]. In addition, a number of clinical and experimental studies have suggested that the topical application of algal sulfated polysaccharides such as fucoidan and sacran improve skin condition and have anti-aging anti-allergic properties [4,5,6].

Sujiaonori, the Japanese name for *Ulva (Enteromorpha) prolifera* Muller, is one of edible marine algae growing in the Kochi prefecture, Japan. We conducted a clinical study that evaluated the effects of daily intake of Sujiaonori algal biomaterial (SBM). We have recently reported that the intake of SBM supplement induced an increase of circulatory adiponectin and improved cardiovascular health in humans [7,8]. Adiponectin, adipocyte-derived anti-inflammatory hormone, is known to prevent a number of cardiovascular and metabolic disorders [9,10,11,12,13]. This hormone and its receptors are also reported to be associated with healthy longevity [14,15]. To our knowledge, there have been no scientific reports on the health of Sujiaonori or its compounds on skin health. This paper reports on the effect of daily intake of a Sujiaonori-based supplement on the skin health of a sample of Japanese women, as well as the anti-allergic effect of Sujiaonori-extracted ulvan (Tosalvan) in vitro.

## 2. Methods

### 2.1. The Effect of SBM on Degranulation of RBL-2H3 Cells and Release of β-Hexosaminidase In Vitro

#### 2.1.1. Agents (Test Compounds) 

Ulvan (algal sulfated polysaccharides) samples used in this study were a gift from the Food Science Laboratory, department of Nutrition, University of Kochi (Kochi, Japan). They were extracted from dried algal samples from Shimanto River, Muroto Sujiaonori Farm (Kochi prefecture) and Yoshino River in the Tokushima prefecture, using the water extraction–alcohol precipitation method, as reported previously. They are sulfate ester-containing polysaccharides made of rhamnose, arabinose, fucose, xylose, glucuronic acid, and glucose [16]. All samples were kept at −20 °C; they were categorized as follows:

(1) **Tosalvan 1**: ulvan samples from Sujiaonori growing in Shimanto River in Kochi, Japan (the name derives from a combination of “**Tosa**”, another popular name for the Kochi prefecture, and **ulvan**);
(2) **Tosalvan 2**: ulvan samples from Sujianori grown at the “Muroto Aonori Farm” in the Kochi prefecture;
(3) **Yoshino SP**: ulvan samples from alga growing in the Yoshino River;
(4) **Control**: modified Tyrode’s buffer (MT-buffer).

#### 2.1.2. Cell Culture

RBL-2H3 cells were maintained in Dulbecco’s Modified Eagle’s Medium (DMEM: Nacalai Tesque, Tokyo, Japan) with 10% (v/v) fetal calf serum (FBS: Sigma-Aldrich, St. Louis, MO, USA), 100 U/mL of penicillin (Nacalai Tesque, Tokyo, Japan), and 100 μg/mL of streptomycin (Nacalai Tesque, Tokyo, Japan) at 37 °C in a humidified atmosphere containing 5% CO_2_. 

#### 2.1.3. β-Hexosaminidase Release Activity

To evaluate IgE-mediated degranulation, a β-hexosaminidase release assay was employed as described previously [16]. RBL-2H3 cells were seeded in a 24-well plate (2.5 × 10^5^ cells/well) in DMEM with 10% FBS and cultured overnight at 37 °C. The cells were then washed twice with PBS (-). They were sensitized with dinitrophenyl (DNP)-specific IgE (Sigma-Aldrich, St. Louis, MO, USA) at 50 ng/mL for 2 h. After the cells were washed with the MT buffer, each ulvan sample (Tosalvan1, Tosalvan2, Yoshino SP) was diluted in the MT buffer, and the concentration used in the experiment was 100 μg/mL. Ten minutes before allergen sensitization, 20 μL of each ulvan solution was added to the culture. After 10 min of incubation, DNP-human serum albumin (HSA) (final concentration 50 ng/mL) was added, and the culture was incubated for 30 min. The supernatant was collected, and the cells were lysed with MT buffer containing 0.1% polyethylene glycol mono-p-isooctylphenyl ether (Triton X100, Wako Pure Chemicals, Osaka, Japan). Aliquots of each supernatant and cell lysate were incubated with 1 mM *p*-nitrophenyl-*N*-acetyl-β-d-glucosamide (Wako Pure Chemicals, Osaka, Japan), solubilized in 0.1 M citrate buffer (pH 4.5) for 30 min at 37 °C. The enzyme reaction was terminated by the addition of 2 M glycine buffer (pH 10.4), and the absorbance was measured at 405 nm. The percentage of β-hexosaminidase release activity by RBL-2H3 cells, after treatment with each agent (ulvan or control product), was calculated using the following equation:

$$Enzyme\;release\;activity\;\left(\%\right)\;\;=\frac{\mathrm{absorption}\;\mathrm{of}\;\mathrm{cell}\;\mathrm{supernatant}}{\mathrm{absorption}\;\mathrm{of}\;\mathrm{cell}\;\mathrm{supernatant}+\mathrm{absorption}\;\mathrm{of}\;\mathrm{cell}\;\mathrm{lysate}}\times 100.$$

### 2.2. The Effect of Daily SBM Intake on Skin Health

#### 2.2.1. Study Design and Subjects

This was a non-randomized investigator-blinded and controlled dietary intervention that evaluated the health effects of daily supplementation of Sujiaonori algal biomaterial (SBM) on adiponectin production, cardiovascular and gastrointestinal health, and skin health. This paper reports the effects of SBM supplementation on transepidermal water loss (TEWL). Details on the methods used in the main study have been published previously [7]. Briefly, participants were divided into two groups: for a total of 28 days, those from the SBM group received 3 g of SBM powder twice a day during meals, whereas controls had to take the same amount of a mixture made of 70% corn starch and 30% spinach powders daily.

#### 2.2.2. Skin Health Testing

Pre- and post-test TEWL values from 29 adult Japanese women who had salivary adiponectin results were analyzed. None of them had an active skin disorder; however, considering high TEWL values in some of those volunteers, the cold weather in winter might have exposed them to xerosis (dry skin), a condition that increases the dermal absorption of chemicals, allergens, etc. and that increases vulnerability to skin disorders such as atopic dermatitis and psoriasis, to some extent. Skin health was assessed using a Cutometer (Courage & Khazaka Co. Ltd, Cologne, Germany) to determine TEWL level, and the test was performed by a dermatologist at baseline and on Day 28 of the study. The measurement was performed two to three times, and the best TEWL value was considered for each of the study subjects. Cutometer dual MPA ( Courage & Khazaka Electronic, Cologne, Germany) (Supplementary Materials Figure S1) is a sensitive device connected to a computer that is placed on the external surface of the left forearm to evaluate the permeability of skin. TEWL is an indicator of skin barrier function and represents the diffusion of water through the stratum corneum. The higher the TEWL, the more permeable and prone to xerosis the skin becomes [17]. 

#### 2.2.3. Ethical Consideration

The ethical approval of the main study protocol was obtained from the ethics committee of the faculty of Nutrition, University of Kochi, Japan (Approval reference: No15-10, November 2015). In addition, this protocol was approved by an international trial registry (registration number: ISRCTN35616776).

### 2.3. Data Collection and Analysis

Baseline and end-of-study data were collected from participants and transcribed on an excel sheet. TEWL data were analyzed by a Student’s t test. For data from the in vitro study, a Tukey’s test was used to compare treatment groups. Stata statistical software (version 14, StataCorp LLC, College Station, TX, USA) was used for data analysis. The statistical significance level was set at a *p*-value (double sided) less than 0.05.

## 3. Results 

### 3.1. Tosalvan Inhibits IgE-Mediated Activation of RBL-2H3 Cells and the Release of β-Hexosaminidase 

**Figure 1:**
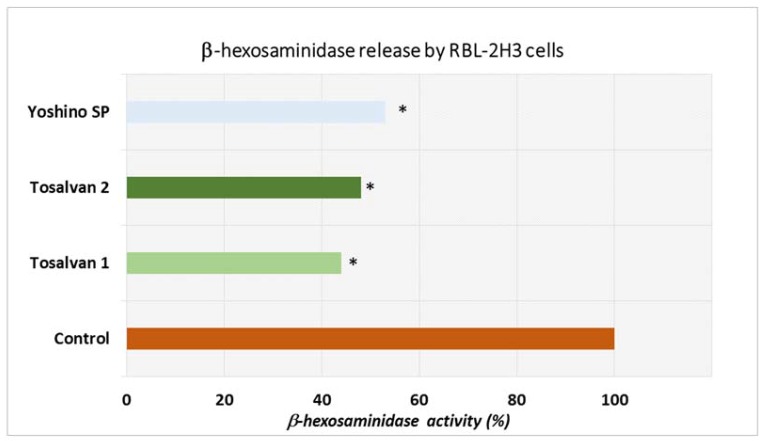
Inhibition of allergen-challenged RBL-2H3 cell activation and release of β-hexosaminidase by Tosalvan from Sujiaonori and Yoshino SP.

**Legend:** SP: sulfated polysaccharide; * *p*-value less than 0.05. The figure shows a marked inhibition of RBL-2H3 cells degranulation and release of β-hexosaminidase by Tosalvan 1/2 and Yoshino SP (*p* < 0.05) (Figure 1).

RBL-2H3 basophilic cells (mast cells) activation has been serving as in vitro experimental model of IgE-mediated skin allergy, with the cell degranulation and the release of markers of allergic inflammation mimic the pathophysiology of allergen-induced skin allergy in vivo. Figure 1 shows a significantly reduced release of β-hexosaminidase by RBL-2H3 cells after allergen challenge in Tosalvan1, Tosalvan2, and Yoshino SP-treated cells, as compared with control cells (*p* < 0.01). Pre-treatment RBL-2H3 cells with Tosalvan1 and Tosalvan2 could lower the release of this allergic inflammatory marker by approximately 50–60%. However, despite the relative better inhibitory effect displayed by Tosalvan1 as compared to Tosalvan2 and Yoshino SP, no significant difference was observed.

### 3.2. Tosalvan-Rich SBM Improves Skin Health through the Reduction of Transepidermal Water Loss (TEWL)

**Figure 2:**
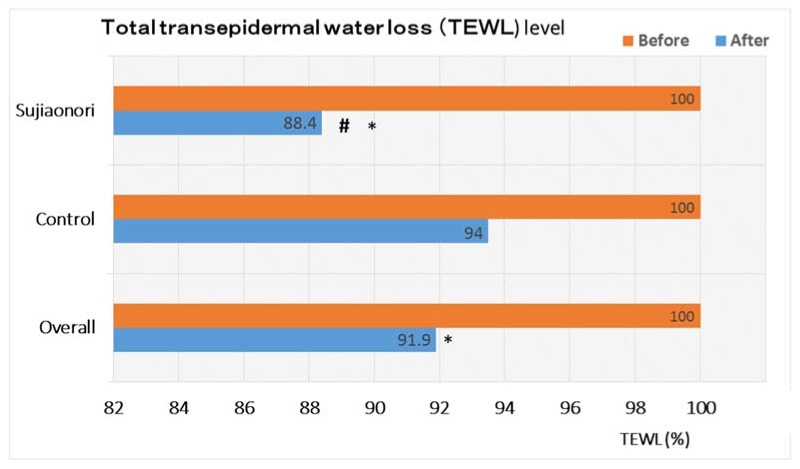
Transepidermal water loss (TEWL; g/m^2^-h) according to the supplementation group (N = 29).

**Legend:** # *p*-value less than 0.001; * *p*-value less than 0.05. The figure shows a reduced TEW level in Sujiaonori (SBM) supplemented group as compared to baseline TEWL (*p* < 0.001) and as compared to the control group (*p* < 0.05) (Figure 2).

As mentioned previously, TEWL is used as one of the markers of skin barrier status and skin health. Figure 2 shows that women supplemented with SBM had a markedly lower TEWL value after a four-week dietary intake of this food product, as compared to the baseline TEWL value (*p* < 0.001), whereas no such a difference was observed in the control group (*p* > 0.05). In addition, when both SBM and control group were compared, a significant difference was observed, with SBM-supplemented women having less TEWL compared with the controls (*p* < 0.05) (Figure 2).

After participants were stratified according to age, a marked reduction in the TEWL level was noted in SBM-supplemented younger women (<30 years of age vs. older ones) at the end of the study (*p* < 0.001) (Figure 2), whereas no significant change in the TEWL value was noted when comparing the same subgroups in the controls (*p* > 0.05).

## 4. Discussion and Conclusions

The present report provides new findings in relation to the bioactive properties of Sujiaonori, a green river alga growing in Japan. Firstly, the in vitro experiment showed that Sujiaonori-derived sulfated polysaccharide, Tosalvan, inhibited the IgE-mediated activation of RBL-2H3 basophilic leukemia cells and the release of β-hexosaminidase, which is one of the markers of allergic reaction. Secondly, daily intake of Sujiaonori biomaterial improved skin barrier status through the reduction of TEWL, a marker of skin permeability, in a sample of Japanese women. 

RBL-2H3 cells are referred to as cells deriving from mast cells, and these cell lines are among the commonly used in vitro models of allergic inflammation both in allergy and immunology research [18,19]. A number of bioactive natural products have been reported to inhibit allergic reaction in vitro and the release of inflammatory markers, such as histamine and β-hexosaminidase, which play an important role in allergic reactions. Our study showed that Tosalvan from *Ulva profilfera*, grown both in rivers and farms in the Kochi prefecture, Japan, as well as ulvans extracted from marine alga growing in the Yoshino River in the Tokushima prefecture inhibited RBL-2H3 cell degranulation and the release of β-hexosaminidase, one of the markers of IgE-mediated allergic inflammation. A study by Chung et al. showed that extracts of *Schizandra chinensis* Baillon exerted anti-allergic activity on IgE-allergen complex-stimulated RBL-2H3 cell line [20]. Recently, Hong et al. have also reported similar findings in a study that evaluated the anti-allergic and anti-inflammatory effects of diterpenoid compounds isolated from the South China Sea marine sponge *Hippospongia lachne* [21]. 

The skin homeostasis depends on a number of factors, such as the permeability of the stratum corneum. In addition, the stratum corneum, which is the most external skin layer, is very dynamic when considering the skin barrier integrity and repair. Its damage induces the loss of intercellular lipid membrane composition and integrity and increases TEWL, a risk factor for skin disorders related to impaired skin barrier such as dry skin and atopic dermatitis [22,23,24]. Recently, researchers in the field of allergic skin diseases have been suggesting the use of substances that enhance the skin barrier function as alternative therapeutic agents for allergic skin disorders.

Several previous works have shown the potential for algal biomaterials and other bioactive compounds to be used as skin barrier enhancers and alternative anti-allergic agents. Ngatu and colleagues have reported that Sacran, a sulfated polysaccharide from river alga *Aphanothece sacrum*, improves skin barrier and exerts anti-allergic activity through the upregulation of filaggrin production [25]. In addition, in a recent Korean study, a randomized clinical trial, a 12-week dietary intake of galacto-oligosaccharides reduced TEWL and improved skin health [26]. Furthermore, Chang and colleagues recently demonstrated that the daily application of a skincare product made of ceramide and filaggrin formulation improved TEWL, dry skin, and other aging skin symptoms in the elderly [27]. In our study, the daily intake of Sujiaonori-based supplement markedly reduced TEWL, suggesting its potential use as a skin health enhancing food product. Moreover, in our study, given that Sujiaonori-derived Tosalvan could attenuate allergen-induced allergic reaction in vitro might possibly corroborate the skin barrier enhancing effect displayed by SBM supplementation in humans. This suggests that Sujiaonori contains biomaterials with a potential to be useful in the prevention and management of allergic skin disorders. Future clinical investigations are needed to confirm this assertion.

This study has limitations. The sample size was relatively small for each of the study groups. Thus, it is advisable to increase the number of participants in future investigations. Another fact is that this study did not explore the mechanism by which the Sujiaonori or its compounds could improve skin health status. Nonetheless, it is known that adiponectin, an adipocyte-specific adipokine involved in the systemic metabolism, plays a role in skin health as it upregulates hyaluronic acid production in the skin [28]. Hyaluronic acid is known as a key molecule for skin moisture, and it has the capacity to bind and retain water molecules in the skin [29,30,31]. We previously reported that the intake of Sujiaonori-based supplement increased circulatory adiponectin [7]. This effect might also be induced locally on skin adipocytes located in the subcutaneous adipose tissue lying beneath the dermis, and adiponectin promotes the production of dermal hyaluronic acid [32], a compound known to enhance the skin barrier function. Thus, given this effect of hyaluronic acid on skin barrier, SBM could have possibly induced the improvement of skin health (TEWL) through its adiponectin secretion upregulating effect. This fact should be investigated in order to confirm the relationship between Sujiaonori, or its bioactive compounds, and skin hyaluronic acid. Furthermore, in vitro experiments that employ different kinds of cell lines involved in allergic reactions and the experiments measure a variety of inflammatory cytokines (Th-1, Th-2) and other markers of allergy should be envisaged in order to explore the anti-inflammatory effects of Sujiaonori biomaterials.

In conclusion, the report showed that bioactive materials from river alga Sujiaonori (Tosalvan and algal supplement) inhibited allergic reaction and improved skin health, respectively. Future investigations are needed to confirm the beneficial effects of Sujiaonori biomaterials on human health.

## Supplementary Materials

The following are available online at www.mdpi.com/2079-4983/8/3/37/s1. Figure S1: Cutometer Dual MPA 580 for TEWL measurement.
