# Supplementary figures and images for: Sujiaonori-Derived Algal Biomaterials Inhibit Allergic Reaction in Allergen-Sensitized RBL-2H3 Cell Line and Improve Skin Health in Humans
Source: J Funct Biomater. 2017 Aug 29;8(3):37. doi: 10.3390/jfb8030037 (PMC5618288; doi:10.3390/jfb8030037)

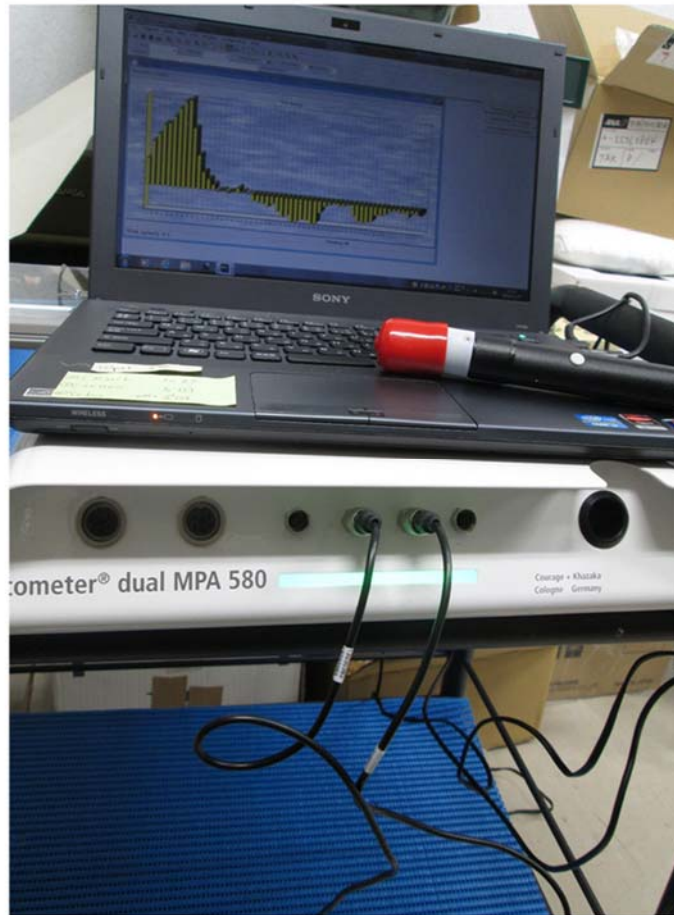

**Supplementary Figure 1.** Cutometer Dual MPA 580 for TEWL measurement.

Supplement: Supplementary file 1 [file jfb-08-00037-s001.pdf]
